# Measuring Neighborhood Walkable Environments: A Comparison of Three Approaches

**DOI:** 10.3390/ijerph14060593

**Published:** 2017-06-03

**Authors:** Yen-Cheng Chiang, William Sullivan, Linda Larsen

**Affiliations:** 1 Department of Landscape Architecture, National Chiayi University, Chiayi 60004, Taiwan; 2 Department of Landscape Architecture, University of Illinois at Urbana-Champaign, Champaign, IL 61820, USA; wcsulliv@Illinois.edu; 3 Department of Rhetoric and Composition Studies, University of Illinois at Urbana-Champaign, Urbana, IL 61801, USA; lflarsen@illinois.edu

**Keywords:** virtual assessment, neighborhood environment, walking, walkability, urban design

## Abstract

Multiple studies have revealed the impact of walkable environments on physical activity. Scholars attach considerable importance to leisure and health-related walking. Recent studies have used Google Street View as an instrument to assess city streets and walkable environments; however, no study has compared the validity of Google Street View assessments of walkable environment attributes to assessments made by local residents and compiled from field visits. In this study, we involved nearby residents and compared the extent to which Google Street View assessments of the walkable environment correlated with assessments from local residents and with field visits. We determined the assessment approaches (local resident or field visit assessments) that exhibited the highest agreement with Google Street View. One city with relatively high-quality walkable environments and one city with relatively low-quality walkable environments were examined, and three neighborhoods from each city were surveyed. Participants in each neighborhood used one of three approaches to assess the walkability of the environment: 15 local residents assessed the environment using a map, 15 participants made a field visit to assess the environment, and 15 participants used Google Street View to assess the environment, yielding a total of 90 valid samples for the two cities. Findings revealed that the three approaches to assessing neighborhood walkability were highly correlated for traffic safety, aesthetics, sidewalk quality, and physical barriers. Compared with assessments from participants making field visits, assessments by local residents were more highly correlated with Google Street View assessments. Google Street View provides a more convenient, low-cost, efficient, and safe approach to assess neighborhood walkability. The results of this study may facilitate future large-scale walkable environment surveys, effectively reduce expenses, and improve survey efficiency.

## 1. Introduction

Public health research has shown that features of neighborhood environments are associated with health behaviors and outcomes. One such feature is perceived walkability. Perceived neighborhood walkability is typically measured through self-reports that include questions related to the built environment regarding residential density, proximity and access to stores and facilities (e.g., land use mix diversity and access), street connectivity, aesthetics, walking facilities, and safety from traffic and crime [1].

Over the past 20 years, studies have connected these neighborhood attributes to how much people walk in their neighborhoods. For example, diverse land use has been shown to enhance people’s willingness to walk [2], while heavy traffic flow made people feel unsafe [3]. A green environment encouraged people to walk [4]. Crime, perceptions of safety, neighborhood disorder, traffic, and other aspects of the social environment are associated with less physical activity among children and adolescents [5].

The public’s willingness to walk depends a great deal on the quality of the walkable environment, and city planners and researchers need accurate and efficient ways of evaluating this quality to assess and improve neighborhood environments. Neighborhood features are commonly inventoried using surveys, administrative data, or observer audits, and each of these strategies has benefits and limitations. For example, self-reports may produce personal biases (e.g., those in poor health are more likely to report poorer neighborhood conditions) [6]. Administrative data refers to information collected by governments and other organizations primarily for administrative purposes such as registration, transaction, and record keeping, usually during the delivery of a particular service (e.g., welfare, health, educational). Some researchers have used administrative data and geographic information systems (GISs) to measure a neighborhood environment. Although some cities release their administrative data, many do not and even when they do, they are often inconsistently available or collected using a variety of methodologies across jurisdictions [7]. The study of neighborhood environments presents practical challenges, especially in studies that use large and geographically dispersed samples. Large-scale walkable environment surveys are both time-consuming and expensive, and weather changes and safety concerns also require consideration. Most neighborhood surveys are confined to relatively small neighborhoods because large-scale surveys are difficult to perform [8]. Some areas with high crime rates can even pose a danger to survey personnel [9].

Google Street View, a tool that provides panoramic views of many streets throughout the world, provides a convenient, fast, low-cost, and safe survey approach to gaining access to the features that impact walkability. Compared to employing trained researchers to walk the neighborhoods under consideration, collecting walkability data via Google Street View saves time and money while providing a 360° street view of many streets around the world. Users can obtain a visual experience of walking on the streets while using their computers.

Using Google Street View makes it easy to obtain data of urban or neighborhood spaces. In recent years, advancing technologies have rendered image updates more efficient, and many studies have used Google Street View as a survey tool to evaluate urban spaces or walkable environments [7,8,10,11].

To our knowledge, however, the validity of using Google Street View for determining neighborhood characteristics is unclear. Although research has demonstrated that field audits and virtual audits by trained professionals yield acceptable correlations, we do not know the extent to which virtual audits and the reports of local residents are correlated. Local residents have been shown to accurately assess local conditions [12]. Thus, in this study, we investigate the extent to which descriptions provided by local residents and assessments from field visits are consistent with Google Street View assessments.

### 1.1. Empirical Studies of Google Street View

Google Street View is a tool available for free in Google Maps and Google Earth that provides 360° panoramic views of many streets throughout the world. It was launched in 2007 in several cities in the United States, and has since expanded to include cities and rural areas worldwide. Following the expansion of service and increase in application examples, a trend of integrating street view images with traffic information was established. Street view images provide an intuitive portrayal of people’s daily living spaces, whereas geographic data systems store real world object attributes and abstract spatial data. The two can be integrated by using various virtual and augmented reality technologies. Thus, street view images form a novel display platform for geographic data.

Still, there are some problems that need to be overcome with these Google tools, such as map updates, an inability to determine real-time flow data (vehicle flow), and perspective problems [10,11]. In spite of these problems, however, Taylor et al. argued that Google Street View is advantageous because it enables efficient environment evaluations and a simultaneous comparison of multiple environment samples. For example, investigators evaluated the environmental attributes of parks and green areas, which took only 4 h with Google Street View but 42 h of on-site evaluation [8]. In terms of time, using Google Street View can dramatically shorten the research process.

In recent years, no consistent results have been obtained in studies comparing Google Street View data with data collected in person in neighborhoods. On the one hand, some large-scale environmental attributes such as land use mix (e.g., residential and industrial) [13,14] and traffic safety [15] exhibit excellent levels of inter-rater reliability. Clarke et al. compared two groups of participants in Chicago, regarding field visits and Google Street View results. The results indicated a strong correlation between the two groups, particularly in recreational facilities, restaurants, and land use [10]. Other results for pedestrian safety (intersections) and motorized traffic from New York City [7], and for variable sporting facilities present in the parks of Sydney [8] also had a high degree of correlation. Vanwolleghem, van Dyck, Ducheyne, de Bourdeaudhuij, and Cardon indicated that the presence of trees and attractive natural features demonstrated moderate agreement [16].

Considering small-scale studies, Ben-Joseph, Lee, Cromley, Laden, and Troped compared the usefulness of the three web tools—Google Maps, Google Street View, and Microsoft Oblique Viewer (Bing Maps)—and determined that Google Street View was the most useful for measuring small-scale features [17]. Some studies found that there was a high agreement between physical and virtual audits of walking surface and walking infrastructure [18]. In addition, some studies have indicated that Google Street View may accurately identify the presence of detailed features in sidewalks (benches, ramps, or curb cuts) [19]. Griew et al. asked two groups to survey the walkable environments in the English town of Wigan by using on-site observations and Google Street View. The results showed relatively low consistency for pavement quality, lighting, and road permeability, but high consistency (>70%) for other detailed attributes such as pavement width and obstructions, and curb paving quality [11].

### 1.2. Research Aim

There are two gaps in our knowledge regarding the use of Google Street View as a tool for helping scholars measure the characteristics of neighborhood environments. First, most previous studies only investigated the correlation between on-site observations and Google Street View data; no study has incorporated the reports of local residents. Because local residents have lived in the area in question, their judgment of the attributes of the surrounding environment should approximate real-life conditions. Second, previous study results have been inconsistent. Some studies found that Google Street View can be used to assess large-scale environmental attributes, whereas other studies reported that Google Street View can be used to assess only small-scale environmental attributes. To address these gaps, we included local residents in a study designed to (a) compare three approaches (local residents, field visits, and Google Street View) to determine the extent to which these approaches correlate regarding walkable environment attributes; and (b) to elucidate whether Google Street View assessments exhibit higher agreement with local resident or field visit assessments.

## 2. Methods

### 2.1. Study Site Selection

**Figure 1:**
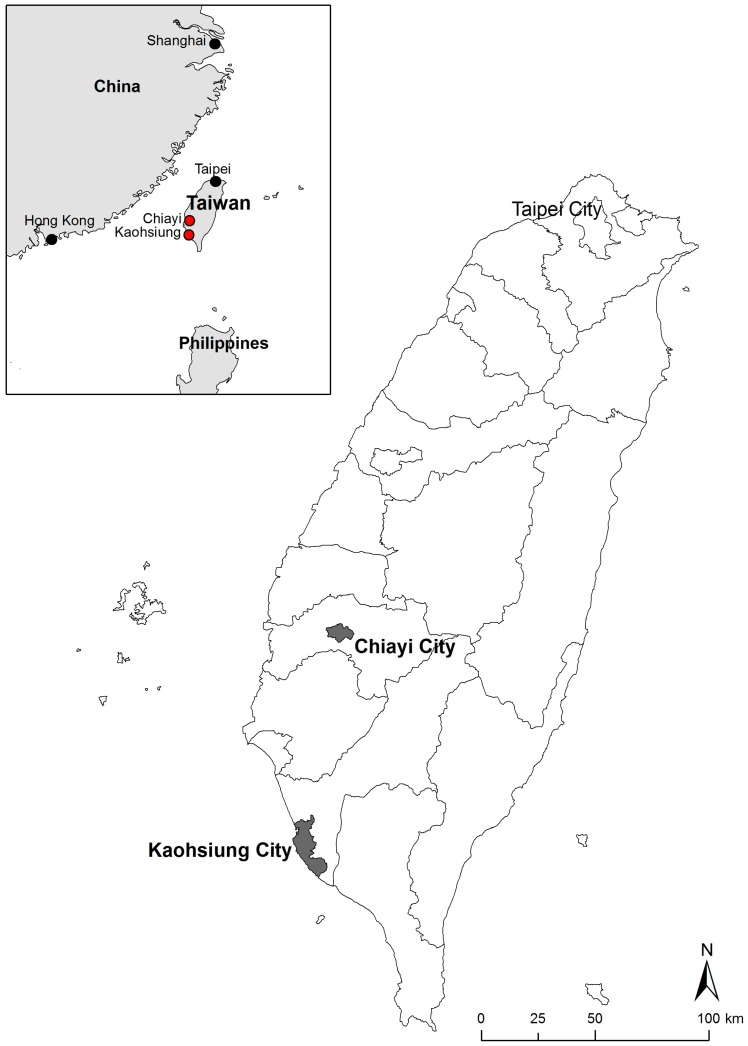
Location of the two study sites (Chiayi City and Kaohsiung City) in Taiwan.

**Figure 2:**
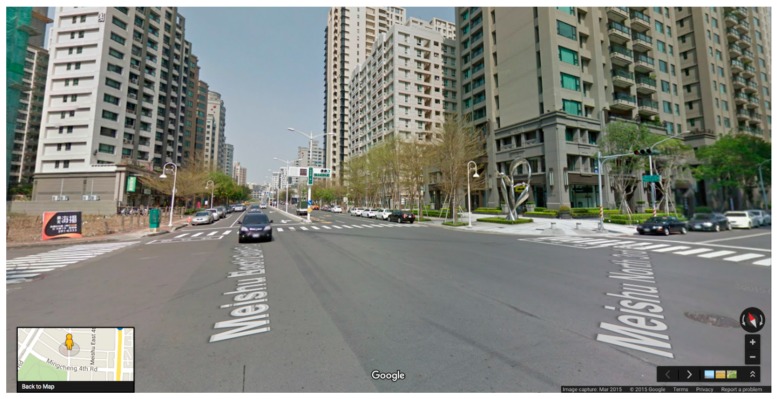
Example of a neighborhood in Kaohsiung City where the walkable environment is relatively good. Source: Google Street View.

**Figure 3:**
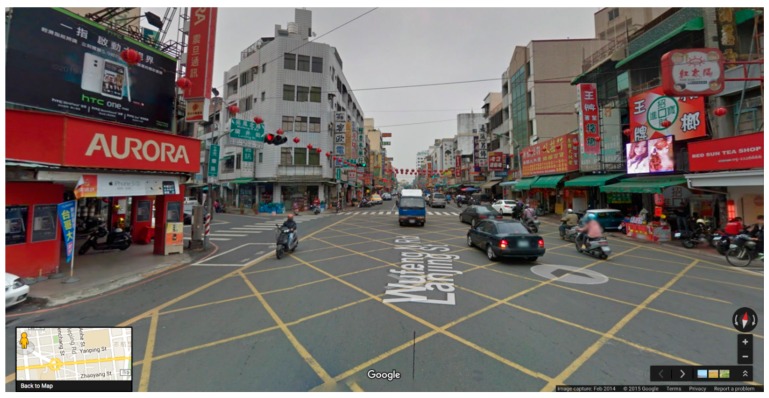
Example of a neighborhood in Chiayi City where the walkable environment is relatively poor. Source: Google Street View.

Due to differences in the quality of their walkable environments, Kaohsiung City (high quality) and Chiayi City (low quality) in Taiwan were selected as the study sites (Figure 1). The Google Street View image data for these two cities were last updated in 2014. In the process of selecting the study neighborhood, we first selected 10 neighborhoods from each city. The neighborhood scope was defined according to Zacharias (2001), who indicated that people can accept a walking distance of 500–1000 m; we adopted a 500 m radius. Three trained researchers conducted field visits to the 20 neighborhoods and evaluated them for the following four walkable environment attributes: (a) Connectivity: the continuity of the walking space network; the more paths and alternative roads that exist, the higher the connectivity is; (b) Sidewalk features and quality: sidewalk width, maintenance, pavement material, and accessibility; (c) Safety: traffic safety (traffic flow volume, traffic signs, and pedestrian crossings) and social safety (graffiti, abandoned houses); and (d) Aesthetics: trees along the roadside, beautiful buildings, public art, and attractive landmarks. The researchers were asked to rate the neighborhoods on a 10-point scale from 1 (worst) to 10 (best). Due to time and cost considerations, after calculating the average rating of each attribute, we selected three neighborhoods with relatively good walkable environments and three neighborhoods with relatively poor walkable environments from each city (Figure 2 and Figure 3).

### 2.2. Participant Training and Survey Procedure

We assessed the walkability of the three neighborhoods using three approaches (local residents, field visits, and Google Street View), and 15 participants were recruited for each approach. A total of 90 participants were recruited. To avoid climate factors influencing the participants’ rating, the local resident and field visit data were collected in April and May 2015, and all surveying was conducted during daytime under pleasant climate conditions. The Google Street View browsing was completed from May to July 2015.

- Local residents: To select people with a thorough understanding of the area’s environmental conditions, we selected residents who had lived in the area for three or more years for a questionnaire survey. We recruited them as they walked on the sidewalks in their neighborhoods. They were not required to make field visits. Instead, they were given a map of the area and asked to draw from their experiences in the neighborhood while completing the questionnaire.
- Field visits: We recruited participants who were walking outdoors in the two cities and who were not residents of the neighborhoods in question. The participants received 1 h of training before commencing the assessment. They were first given a map of the area and the travel route and scope of their activities were explained. To prevent discussions among the participants from influencing the results, only one person performed the assessment at a time. The participants were asked to visit every street and alley in a 500 m radius of a center point in the neighborhood. They were asked to walk at their normal walking speed (about 5.0 km/h; 3.1 mph) [20], experience the environment, and then complete the questionnaire.
- Google Street View assessment: We recruited participants by putting up recruitment posters at a university campus. Before the assessment, the participants received 2 h of training, during which they were informed about the browsing operation mode, route, scope, and browsing speed. They were asked to browse each street and alley by viewing Google Street images of the neighborhood before completing the questionnaire.

### 2.3. Walkability Attributes

This study aimed to elucidate the walkable environment attributes in the studied cities. Therefore, eight categories (street connectivity, social safety, traffic safety, aesthetics, sidewalk quality, physical barriers, amenities, and others) were adopted from Griew et al. [11] and Rundle et al. [7] as walkability factors. Certain categories that could not be immediately measured in Google Street View were excluded, such as changes between daytime and nighttime, perspective problems, and climate [7,8]. All categories and attributes are listed in Table 1.

### 2.4. Data Analysis

To investigate the inter-rater reliability (local residents, field visits, and Google Street View), we used a two-way mixed model intraclass correlation coefficient (ICC) to perform our analyses. Following the classification of Landis and Koch, the cutoff ranges for the ICC values were 0.0–0.20 for weak agreement, 0.21–0.40 for poor agreement, 0.41–0.60 for moderate agreement, 0.61–0.80 for substantial agreement, and 0.81–1.00 for almost perfect agreement [21]. IBM SPSS Version 22.0 (Armonk, NY, USA) was used for all data analyses.

### 2.5. Ethical Statement

All subjects gave their informed consent for inclusion before they participated in the study. The study was conducted in accordance with the Declaration of Helsinki, and the protocol was approved by the Research Ethics Committee for Human Behavioral Sciences of National Cheng Kung University, Taiwan (#102-134).

## 3. Results

### 3.1. Participant Demographics

Among the 90 participants, 51.1% were men and 34.4% were aged 26–35 years. The participants in the local residents group had lived in the study area for an average of 6.2 years (SD = 2.7) (Table 2).

### 3.2. Inter-Rater Reliability of the Walkability Categories

To what extent did the assessments of walkability obtained from local residents and field visits correlate with those obtained from Google Street View? To answer this question, we examined the ICC among the eight categories for each of the three approaches. As can be seen in Table 3, assessments obtained from both local residents and field visits were in substantial or nearly perfect agreement with Google Street View for more than half of the walkability categories (traffic safety, aesthetics, sidewalk quality, and physical barriers). Assessments made by local residents regarding street connectivity were also strongly correlated with Google Street View. For other walkability categories (e.g., amenities and others), there was only slight agreement between local residents and Google Street View, or between field visits and Google Street View.

### 3.3. Inter-Rater Reliability of the Walkability Attributes

To what extent were the assessments made by the three methods consistent? To address this question, we examined the ICC for each pair of assessments and found the correlations to be similar. As can be seen in Table 4, the following attributes were consistent: alternative paths, road safety, beautiful views in the surroundings, attractive scenery, roadside plantings, roadside trees, sidewalk width, pavement smoothness, scooters occupying the sidewalk, rain shelters, benches, and street signs. Intriguingly, the local residents and Google Street View approaches showed substantial agreement regarding intersections; however, assessments from field visits and Google Street View exhibited poor agreement regarding intersections (ICC = 0.39). Regarding the cul-de-sac attribute, assessments made by local residents and Google Street View data exhibited only slight agreement, whereas field visits and Google Street View data exhibited nearly perfect agreement (ICC = 0.80).

The local resident and Google Street View approaches showed moderate agreement for seven out of the 27 attributes (graffiti, abandoned houses or cars, pedestrian flow volume, vehicle flow volume, traffic signs, street vendors occupying the sidewalk, and cul-de-sac). Field visits and Google Street View data exhibited moderate agreement for three of the 27 attributes (traffic signs, benches, and accessibility ramps). Among the three approaches, weak agreement (ICC < 0.20) and poor agreement (ICC = 0.20–0.40) were observed for shop window decoration, lighting, and bus stops.

## 4. Discussion

This study examined the extent to which local resident assessments and field visit assessments correlated with assessments made using Google Street View. We found that assessments made by both local residents and participants during field visits correlated with assessments made using Google Street View. In particular, four of the walkability categories (traffic safety, aesthetics, sidewalk quality, and physical barriers) were consistent with one another. One category, aesthetics, showed almost perfect agreement. These results are similar to those of previous studies [14,18]. Moreover, although assessments of street connectivity made by local residents and participants using Google Street View were in agreement, assessments from field visits and Google Street View were weakly correlated. In the categories of amenities and others, certain installations such as benches and street signs, were often overlooked because of their small size.

In particular, we found that assessments from local residents and field visits correlated with Google Street View assessments for many of the walkability attributes. The 13 environmental attributes (alternative paths, road safety, beautiful views in the surroundings, attractive scenery, roadside plantings, roadside trees, sidewalk width, pavement smoothness, sidewalk cleanness, scooters occupying the sidewalk, rain shelters, benches, and street signs) attained the highest correlations. In other words, the agreement among the three approaches was highest for these attributes. These results indicate that, compared with detailed features (graffiti, shop windows, lighting, and accessibility ramps), relatively large-scale attributes (e.g., alternative paths) and features (e.g., attractive scenery, planted trees, and planted flowers) attained relatively higher agreement. Therefore, we infer that relatively large-scale attributes and features (e.g., roadside plantings and road safety) would directly influence walkability, whereas detailed features (e.g., graffiti and shop windows) had a relatively insignificant impact.

The level of agreement among the three assessment approaches differed only for intersections and cul-de-sac attributes. Correlations between local resident assessments and Google Street View assessments for intersections were higher than those between field visits and Google Street View assessments. This result may be because the local residents are more familiar with the intersections in their neighborhood than the participants conducting field visits. Participants completing the Google Street View assessment could browse freely and thus were able to gain a clear impression of the intersections. This may account for the level of agreement between the Google Street View and local resident approaches.

For the cul-de-sac assessment, data from the field visits were more in agreement with the Google Street View assessment, and assessments from local residents and Google Street View were less in agreement. Field visits and Google Street View may have offered participants a greater opportunity to explore the setting (in reality or in the simulations), giving them a deeper impression of where the dead end streets were located.

Several environmental factors did not attain a high level of agreement. Not surprisingly, temporary and dynamic factors related to pedestrian flow volume, vehicle flow volume, and street vendors occupying the sidewalk were all relatively difficult to investigate using Google Street View. Furthermore, some detailed features on the street may have been difficult to observe with Google Street View (e.g., graffiti, store window decorations, distinctive business signs, accessibility ramps, and bus stops). Each click on Google Street View may cause a 5- to 10-m jump, making some environmental details impossible to continuously assess.

Overall, the results of this study support the feasibility of using Google Street View to assess the walkability of neighborhood environments. Using Google Street View was a convenient, low-cost, efficient, and safe approach. These findings are consistent with the results of previous studies [7,15,22]. We conclude that Google Street View is a reliable tool for measuring the contextual attributes of streets and neighborhood environments.

Based on the results of our study, we offer the following recommendations for practitioners:

- Assessing a site by using Google Street View will be adequate when looking at large-scale environmental attributes such as street connectivity (i.e., intersections and alternative paths). Employing Google Street View is an efficient and cheap way of assessing the aesthetics of a site.
- Gaining residents’ feedback is crucial for aspects such as street connectivity and traffic safety, because residents are familiar with their neighborhood environment and traffic conditions.
- Regarding the reliability of local residents and Google Street View, some detailed attributes (e.g., graffiti, abandoned houses or cars, traffic signs, and dead end streets) and some dynamic information categories (e.g., pedestrian and vehicle flow volume) were in moderate agreement; however, local residents and Google Street View were more reliable than field visits.
- When residents’ feedback is not feasible, field visits can provide correct information about the sidewalk quality and physical barriers of specific sites.

## 5. Limitations

Although the results of this study show that Google Street View attained acceptable agreement with local resident and field study approaches for many environmental attributes, this study still has several limitations.

First, temporary and dynamic data (e.g., vehicle speed, vehicle flow volume, and street vendor commercial activities) cannot be evaluated; it is impossible for Google Street View to accurately indicate factors perceived by the senses (e.g., temperature changes, noise, and exhaust gas emissions) [23]. Moreover, street block size and building height are difficult to assess with Google Street View.

Second, most Google Street View images were shot in the center of the road, causing many detailed features surrounding the sidewalks such as accessibility ramps, lighting, and shop window decorations to be ignored. Rundle et al. also found that features such as pavement quality and width, and nighttime lighting generally cannot be observed using Google Street View [7]. In addition, assessing sidewalk details becomes more difficult when vehicles are parked by the roadside. Therefore, to improve the agreement between the Google Street View and field visit assessments, weather and time conditions should be selected that match those at the time of the Google Street View shooting. It would also be helpful to know whether the Google Street View images were captured during peak or non-peak hours so that the field visits could be conducted at similar times. Furthermore, Google Street View does not allow for elevation and gradient, ambience, and how people use spaces.

Third, it is also important to consider whether too much time has passed since the street view images were captured. A previous study suggested that if the images are more than three years old, the environmental evaluation can be affected [8]. In this study, the assessments were completed in the same year that the Google Street View images were captured. Thus, this was not a limitation for the present study.

Fourth, local residents were only shown maps and had to rely on their personal memories and user habits for their assessments, which are not always reliable. Furthermore, many of the participants were between the ages of 26 and 35; the lack of older or younger participants [24,25], who are also frequent users of urban spaces, may have led to biased results. Future research should include a wider range of users to achieve more credible Google Street View ratings.

## 6. Conclusions

Google Street View virtual technologies have enabled the virtual exploration of the world. Since environmental assessments from Google Street View are free, easy, and less time-intensive than field visits or other methods, Google Street View has created new opportunities for conducting international comparative research on built environments [8,11,18]. It can be used to compare different areas and increase the surveyed area to gain greater understanding. However, are environmental assessments using Google Street View reliable when compared to field visits or assessments from local residents? Our research suggests that there is strong agreement between these different assessments, especially when assessing large-scale aspects of the environment and aesthetics. Designers and planners should use caution when using Google Street View to assess small features in an environment or temporal aspects of the environment. Field visits or assessments from local residents may be preferred in those circumstances. Our results suggest that Google Street View is an efficient tool for understanding neighborhood environments and city street conditions, and can be adopted to enable policy makers to understand the relationships between environmental attributes, urban design, and public health.

## Figures and Tables

**Table 1:** Walkability measure instruments.

Categories	Attributes	Levels
Street connectivity	Intersections	1 (very few) to 5 (numerous)
	Alternative paths	1 (very few) to 5 (numerous)
Social safety	Graffiti	1 (common) to 5 (none)
	Abandoned houses or cars	1 (common) to 5 (none)
	Pedestrian flow volume	1 (very few) to 5 (numerous)
	Security of the surroundings	1 (very unsafe) to 5 (very safe)
Traffic safety	Vehicle flow volume	1 (very high) to 5 (very low)
	Road safety	1 (unsafe) to 5 (safe)
	Traffic signs	1 (very insufficient) to 5 (very sufficient)
Aesthetics	Beautiful views in the surroundings	1 (none) to 5 (common)
	Attractive scenery	1 (none) to 5 (common)
	Shop window decoration	1 (none) to 5 (common)
	Roadside plantings	1 (none) to 5 (common)
	Roadside trees	1 (none) to 5 (common)
	Distinctive business signs	1 (none) to 5 (common)
Sidewalk quality	Sidewalk width	1 (very insufficient) to 5 (very sufficient)
	Pavement smoothness	1 (very coarse) to 5 (very smooth)
	Sidewalk cleanness	1 (very unclean) to 5 (very clean)
Physical barrier	Scooters occupying the sidewalk	1 (common) to 5 (none)
	Street vendors occupying the sidewalk	1 (common) to 5 (none)
	Cul-de-sac	1 (common) to 5 (none)
Amenities	Rain shelters	1 (none) to 5 (common)
	Benches	1 (none) to 5 (common)
	Lighting	1 (none) to 5 (common)
Others	Accessibility ramps	1 (none) to 5 (common)
	Bus stops	1 (none) to 5 (common)
	Street signs	1 (none) to 5 (common)

**Table 2:** Demographic information of the participants (*n* = 90).

Variable	*n* (%)
Gender	
Male	46 (51.1)
Female	44 (48.9)
Age (years)	
≤18	1 (1.1)
19–25	23 (25.6)
26–35	31 (34.4)
36–45	4 (4.4)
46–64	24 (26.7)
≥65	7 (7.8)

**Table 3:** ICC for the walkability categories.

Categories	ICC	
	Local Residents vs. Google	Field Visits vs. Google
Street connectivity	0.73 ^d^	0.20 ^b^
Social safety	0.16 ^a^	0.19 ^a^
Traffic safety	0.76 ^d^	0.73 ^d^
Aesthetics	0.85 ^e^	0.81 ^e^
Sidewalk quality	0.67 ^d^	0.73 ^d^
Physical barrier	0.68 ^d^	0.72 ^d^
Amenities	0.53 ^c^	0.40 ^c^
Others	0.33 ^b^	0.42 ^c^

Notes: ICC = intraclass correlation coefficient. ^a^ Weak agreement (ICC < 0.2); ^b^ Poor agreement (ICC = 0.2–0.4); ^c^ Moderate agreement (ICC = 0.4–0.6); ^d^ Substantial agreement (ICC = 0.6–0.8); ^e^ Almost perfect agreement (ICC > 0.8).

**Table 4:** ICC for the walkability attributes.

Categories	Attributes	ICC	
		Local Residents vs. Google	Field Visits vs. Google
Street connectivity	Intersections	0.73 ^d^	0.39 ^b^
	Alternative paths	0.87 ^e^	0.82 ^e^
Social safety	Graffiti	0.53 ^c^	0.24 ^b^
	Abandoned houses or cars	0.47 ^c^	0.05 ^a^
	Pedestrian flow volume	0.57 ^c^	0.21 ^b^
	Security of the surroundings	0.22 ^b^	0.15 ^a^
Traffic safety	Vehicle flow volume	0.50 ^c^	0.11 ^a^
	Road safety	0.78 ^d^	0.63 ^d^
	Traffic signs	0.57 ^c^	0.45 ^c^
Aesthetics	Beautiful views in the surroundings	0.87 ^e^	0.82 ^e^
	Attractive scenery	0.87 ^e^	0.88 ^e^
	Shop window decoration	0.32 ^b^	0.33 ^b^
	Roadside plantings	0.85 ^e^	0.79 ^d^
	Roadside trees	0.87 ^e^	0.93 ^e^
	Distinctive business signs	0.25 ^b^	0.39 ^b^
Sidewalk quality	Sidewalk width	0.78 ^d^	0.83 ^e^
	Pavement smoothness	0.83 ^e^	0.78 ^d^
	Sidewalk cleanness	0.89 ^e^	0.88 ^e^
Physical barrier	Scooters occupying the sidewalk	0.83 ^e^	0.68 ^d^
	Street vendors occupying the sidewalk	0.44 ^c^	0.24 ^b^
	Cul-de-sac	0.52 ^c^	0.80 ^e^
Amenities	Rain shelters	0.65 ^d^	0.80 ^e^
	Benches	0.86 ^e^	0.60 ^d^
	Lighting	0.14 ^a^	0.36 ^b^
Others	Accessibility ramps	0.38 ^b^	0.40 ^c^
	Bus stops	0.38 ^b^	0.29 ^b^
	Street signs	0.78 ^d^	0.63 ^d^

Notes: ICC = intraclass correlation coefficient. ^a^ Weak agreement (ICC < 0.2); ^b^ Poor agreement (ICC = 0.2–0.4); ^c^ Moderate agreement (ICC = 0.4–0.6); ^d^ Substantial agreement (ICC = 0.6–0.8); ^e^ Almost perfect agreement (ICC > 0.8).

## Abbreviations

The following abbreviations are used in this manuscript:

ICC: intraclass correlation coefficient
